# Prevalence of comorbidities post mild traumatic brain injuries: a traumatic brain injury model systems study

**DOI:** 10.3389/fnhum.2023.1158483

**Published:** 2023-06-15

**Authors:** Shyam Kumar Sudhakar, Shreya Sridhar, Satvika Char, Kathan Pandya, Kaustav Mehta

**Affiliations:** Division of Sciences, School of Interwoven Arts and Sciences, Krea University, Sri City, India

**Keywords:** traumatic brain injury, comorbidities, prevalence, psychiatric, mild TBI, rehabilatation, medical complications

## Abstract

Traumatic brain injury (TBI) is associated with an increased risk of long-lasting health-related complications. Survivors of brain trauma often experience comorbidities which could further dampen functional recovery and severely interfere with their day-to-day functioning after injury. Of the three TBI severity types, mild TBI constitutes a significant proportion of total TBI cases, yet a comprehensive study on medical and psychiatric complications experienced by mild TBI subjects at a particular time point is missing in the field. In this study, we aim to quantify the prevalence of psychiatric and medical comorbidities post mild TBI and understand how these comorbidities are influenced by demographic factors (age, and sex) through secondary analysis of patient data from the TBI Model Systems (TBIMS) national database. Utilizing self-reported information from National Health and Nutrition Examination Survey (NHANES), we have performed this analysis on subjects who received inpatient rehabilitation at 5 years post mild TBI. Our analysis revealed that psychiatric comorbidities (anxiety, depression, and post-traumatic stress disorder (PTSD)), chronic pain, and cardiovascular comorbidities were common among survivors with mild TBI. Furthermore, depression exhibits an increased prevalence in the younger compared to an older cohort of subjects whereas the prevalence of rheumatologic, ophthalmological, and cardiovascular comorbidities was higher in the older cohort. Lastly, female survivors of mild TBI demonstrated increased odds of developing PTSD compared to male subjects. The findings of this study would motivate additional analysis and research in the field and could have broader implications for the management of comorbidities after mild TBI.

## Introduction

Traumatic brain injury (TBI) constitutes one of the major global health challenges across the world (Gururaj, 2002; Langlois et al., 2006; Lawrence et al., 2016; Rao et al., 2018). TBI is known to be associated with several health-related complications both in the acute and chronic phases and progressively affects the quality of life of the patient population (Brooks et al., 1986; Shoumitro et al., 1999; Hibbard et al., 2000). Treating TBI and TBI-related comorbidities are challenging to the healthcare systems given the number of people affected in civilian and military settings and the myriad of symptoms and comorbidities that ensue following the injury (Brooks et al., 1986; Hibbard et al., 2000; Gururaj, 2002; Langlois et al., 2006; Dean and Sterr, 2013; Lawrence et al., 2016; Danna-Dos-Santos et al., 2018). As a result, TBI and associated comorbidities could impose a severe financial burden on both the patient population and governments worldwide (Gururaj, 2002; Langlois et al., 2006; Kayani et al., 2009; Lee et al., 2021). Furthermore, numerous clinical trials to find a cure for TBI have failed to produce the desired benefit in the patient population (Ye et al., 2009; Stein, 2015; Sudhakar et al., 2019; Sudhakar, 2023). Existing treatment options focus on preventing secondary brain damage through careful clinical management of patients (Jacobi et al., 2002; Armitage-Chan et al., 2007; Flower and Hellings, 2012; Vella et al., 2017; Dash and Chavali, 2018; Ripley et al., 2019).

TBI can be categorized as mild, moderate, and severe injury types based on the scores of the patients reported using the Glasgow coma scale (GCS) (Robert Laskowski et al., 2015; Jain and Iverson, 2022). Mild TBI can occur as a result of concussion in sports activities, falls in elderly subjects, injuries on the battlefield, and whiplash injuries (Kraus and McArthur, 1996; Cassidy et al., 2004; Rutland-Brown et al., 2006; Robert Laskowski et al., 2015). While moderate-severe TBIs are responsible for a majority of death and disability (Kraus and McArthur, 1996; Cassidy et al., 2004; Rutland-Brown et al., 2006; Robert Laskowski et al., 2015), it has been reported that the incidence of mild TBI is higher compared to moderate-severe ones (Langlois et al., 2006). In addition to that, the incidence of mild TBI cases is often underreported in the literature because these estimates often do not encompass subjects who don’t seek medical attention or in-patient hospitalization (Robert Laskowski et al., 2015). Mild TBI has often been termed a “silent epidemic” because the sign and symptoms of the injury don’t usually manifest immediately leading to difficulties in diagnosis. Understanding the long-term health complications (Dean and Sterr, 2013; Danna-Dos-Santos et al., 2018) in the mild TBI population could lead to better disease management in the absence of any visible problems immediately after the injury (Robert Laskowski et al., 2015). Further adding to the woes of clinical TBI is the heterogeneity of injury types. Heterogeneity is an inherent component of TBI owing to several factors including the type and severity of the injury, the affected brain region(s), different pathoanatomical correlates, illness history, age, and sex, etc (Saatman et al., 2008; Maas, 2016). Heterogeneity is also widespread in mild TBI where distinct clusters of the patient population were reported across multiple datasets based on demographics, injury-related information, and laboratory metrics (Si et al., 2018a,b; Pugh et al., 2021). In addition to an incomplete understanding of the activation of cellular and molecular cascades post mild TBI (Werner and Engelhard, 2007; Schwab et al., 2022), the presence of widespread heterogeneity (Saatman et al., 2008; Maas, 2016; Si et al., 2018a,b) has made it quite challenging to come up with a drug to stop secondary brain injuries from happening (Ye et al., 2009) resulting in low success rates of numerous clinical trials (Ye et al., 2009; Stein, 2015; Sudhakar, 2023). When effective therapeutic options become limited for TBI, properly planned patient care (Vella et al., 2017; Dash and Chavali, 2018) along with proactive disease prevention measures could vastly improve patient experiences.

Several research studies have indicated that individuals with TBI exhibit an increased risk of developing a plethora of medical and mental-health comorbidities (Bryant et al., 2010; Bryant, 2011; Yeh et al., 2013; Jain et al., 2014; Katzenberger et al., 2015; Hammond et al., 2019; Stein et al., 2019). These conditions which severely affect the life of the patients fall under a wide umbrella of diseases that include the following: psychiatric and neurological impairments (Bryant et al., 2010; Bryant, 2011; Yeh et al., 2013; Jain et al., 2014; Hammond et al., 2019; Stein et al., 2019), cardiovascular ailments (Liao et al., 2014; Hammond et al., 2019), gastrointestinal (Katzenberger et al., 2015), and lastly those diseases that interfere with endocrine functioning (Tanriverdi et al., 2008). While these studies are very informative in providing information about the post-traumatic development of comorbidities, systematic studies exploring the prevalence of various health-related comorbidities at a discrete time point(s) post injury are required in the field. A previous TBIMS study quantified the prevalence of TBI comorbidities at a 10-year follow-up time period following the index injury (Hammond et al., 2019). This study elegantly reports the prevalence of 44 comorbidities collected using a survey method called Medical and Mental Health Comorbidities Interview (MMHCI) (Hammond et al., 2019). MMHCI was modelled based on National comorbidity survey replication for the mental health conditions and National Health and Nutrition Examination Survey (NHANES) (Center for Disease Control, National Center for Health Statistics, 1999) and was administered to a section of subjects in the TBI Model Systems (TBIMS) national database (Traumatic Brain Injury Model Systems Program, 2021), a neurotrauma registry consisting of longitudinal patient data collected from multiple trauma centres in the United States. While the study was very informative in reporting the prevalence of a list of 44 comorbidities for individuals with moderate-severe TBI, there is a larger need in the field to perform a similar analysis for subjects with mild TBI and understand how the prevalence of comorbidities post brain trauma depends on demographics and injury-specific variables. Our view was emphasized in a recent review of research studies (Tso et al., 2021) that have utilized the TBIMS database (Traumatic Brain Injury Model Systems Program, 2021), where the authors have pointed out that the comorbidity analyses that were performed using the database are quite sparse and the subject remains relatively unexplored.

We in this study employ a multidimensional approach in quantifying the prevalence of 26 comorbidities at 5- years post mild TBI. Using TBIMS national database (Traumatic Brain Injury Model Systems Program, 2021), we estimated the prevalence of comorbidities as a function of demographic variables (age and sex) from the data of subjects who received inpatient rehabilitation. Our results indicate individuals with mild TBI are confronted with various health-related ailments the prevalence of which is also influenced by age and sex, the two demographic variables included in the analysis.

## Materials and methods

We performed an extensive analysis of patient data using the TBIMS national database (Traumatic Brain Injury Model Systems Program, 2021) and quantified the prevalence of medical and mental health comorbidities at 5-years post mild TBI. In addition to that, we quantify the prevalence of comorbidities as a function of the age and biological sex of the subjects. The analysis of various psychiatric and medical comorbidities was performed using the public version of the TBIMS national database (Traumatic Brain Injury Model Systems Program, 2021). A request to access the public version of the database was placed in November 2021 and the request was approved a few days later (Traumatic Brain Injury Model Systems Program, 2021). Altogether, the TBIMS database contains information about 17,932 subjects (Traumatic Brain Injury Model Systems Program, 2021). Information about the comorbidities was collected using the National Health and Nutritional Examination Survey (NHANES) (Center for Disease Control, National Center for Health Statistics, 1999) where the subjects were asked a series of questions regarding the presence/absence of each comorbidity and their onset (before, at the same time, and after the index injury) at multiple follow up time point (1, 2, 5, 10, 15, 20, and 25 years). If a particular condition was found to be positive at the previous administration, the same item was refrained from asking again. Self-diagnosis was not accepted and only diagnosis originating from a doctor or a certified healthcare professional was included in the database. More information on NHANES administration can be found in the TBIMS data dictionary (Traumatic Brain Injury Model Systems Program, 2021). The public version of the database (Traumatic Brain Injury Model Systems Program, 2021) has information about 26 medical and mental health comorbidities of the subjects at multiple follow-up time points (Traumatic Brain Injury Model Systems Program, 2021). In this study, only data collected at 5-year follow-up was considered for analysis.

The database (Traumatic Brain Injury Model Systems Program, 2021) consists of subjects with a minimum age of 16 years and hence the same inclusion criteria were applied to our analysis. We grouped the subjects into three categories based on the severity of the index TBI: mild, moderate, and severe. The severity of TBI was determined using the Glasgow coma scale (GCS) (Jain and Iverson, 2022). All analyses in this study were performed on mild TBI subjects whose GCS scores fell between 13 and 15 (Jain and Iverson, 2022). Data analysis was performed using the Python programming language. Custom Python scripts were written and executed using the Google Collaboratory application (https://colab.research.google.com/). Patient data was read using the pandas python library (https://pandas.pydata.org/) and analysis of the prevalence of the comorbidities was performed thereafter. All scripts used in the study and data generated as a result of this study will be made available to the scientific community upon request.

### Statistical analysis

All statistical analyses in this study were performed on mild TBI subjects. For each comorbidity, we computed the total number of mild TBI subjects who have the condition with onset at the same time or after the index injury at 5-year follow-up time point. Next, the percentage of subjects with a specific comorbidity was computed as the ratio of the number computed in the previous step to the total number of subjects with and without the condition. This number represents the observed prevalence of each comorbidity at a given follow-up time point (Hammond et al., 2019). We also computed the percentage of subjects with the condition and onset before the index TBI in the same manner. We computed the prevalence using two different descriptions of onset so as to provide maximum information to the medical practitioners as each of these definitions could provide vital insights for designing foresighted patient care programs.

We established statistical significance between the prevalence of comorbidities for young vs old subjects, and male vs female subjects. Subjects whose age at follow-up (5-years post TBI) is less than or equal to 50 years were categorized as young and those with age at follow-up greater than 50 years were categorized as old. For estimating the statistical significance of young vs old subjects, we first computed the odds ratio (McHugh, 2009; Steven and Hoffman, 2022) using the following formula,


(1) $$O\,d\,d\,s\,r\,a\,t\,i\,o\,\left(O\,R\right)=\frac{a^{*}\,d}{b^{*}\,c}$$

where ‘a’ represents the percentage of cases (prevalence) with the comorbidity in the older cohort at 5-year follow-up, ‘b’ represents the percentage of cases in the older cohort without the comorbidity at the same follow-up time period. Similarly, ‘c’ represents the percentage of cases (prevalence) with the comorbidity in the younger cohort at 5-year follow-up and ‘d’ represents the percentage of cases in the younger cohort without the comorbidity at the same follow-up time period. This ratio represents the odds of observing a comorbidity in older subjects compared to the odds in the younger cohort. Similarly, the odds ratio capturing the relative prevalence in female vs male subjects in the cohort was computed to determine the sex dependence of post-traumatic prevalence of comorbidities. After computing the odds ratio, statistical significance was established using Fisher’s-Exact test (95% confidence interval) (McHugh, 2009). The probability ‘*p*’ for Fisher’s-Exact test was calculated using the following formula,


(2) $$p=\frac{\left(a+b\right)!\,\left(c+d\right)!\,\left(a+c\right)!\,\left(b+d\right)!}{a!\,b!\,c!\,d!\,n!}$$

where a, b, c, and d represent the same metrics as mentioned above for the odds ratio and n represents the sum of all four variables. Confidence intervals (McHugh, 2009; Steven and Hoffman, 2022) for the odds ratio were computed using the below formula,


$$u=e^{\left[\ln\left(O\,R\right)+1.96^{*}\,\sqrt{\left(\frac{1}{a}+\frac{1}{b}+\frac{1}{c}+\frac{1}{d}\right)}\right]}$$

(3) $$l=e^{\left[\ln\left(O\,R\right)-1.96^{*}\,\sqrt{\left(\frac{1}{a}+\frac{1}{b}+\frac{1}{c}+\frac{1}{d}\right)}\right]}$$

where u and l are upper and lower bounds of the confidence interval.

To determine if a confounding effect (Jager et al., 2008; Jose et al., 2008) of age at follow-up or effect modification was present in the association between the prevalence and biological sex of the subjects, the following procedure was followed: Two strata were created representing subjects <= 50 years of age and subjects > 50 years of age at 5-year follow-up corresponding to two different values of the confounder. For each stratum, we calculated the odds ratio of the relative prevalence of comorbidities in female subjects compared to male subjects (OR_1_ and OR_2_). We next determined if the stratum-specific odds ratios (OR_1_ and OR_2_) were similar to each other using the Breslow-Day-Tarone test (BDTT) for homogeneity (95% confidence interval) (Breslow and Day, 1980; Breslow, 1996). If the odds ratios were found to be significantly different from each other (*p* < 0.05), we report the stratum-specific odds ratio for the comorbidity in question (effect modification).

If the odds ratios were similar (*p* >= 0.05), we utilized the Mantel-Haenszel test (MHT) (Kuritz et al., 1988; Jose et al., 2008; Tripepi et al., 2010) to determine if there is an association between sex and prevalence of the comorbidity in question (95% confidence interval). Additionally, we report Mantel-Haenszel odds ratio (OR_MH_) (Kuritz et al., 1988; Jose et al., 2008; Tripepi et al., 2010) adjusted for age at follow-up (and magnitude of confounding) which is calculated from the stratum-specific numbers using the following formula,


(4) $$O\,R_{M\,H}=\frac{\sum \frac{a_{i}*d_{i}}{n_{i}}}{\sum \frac{b_{i}*c_{i}}{n_{i}}}$$

where a_*i*_, b_*i*_, c_*i*_, and d_*i*_ represent the same as explained above (for odds ratio) in the ith stratum of the confounder (age at follow-up) and n_*i*_ is computed by adding all four variables a_*i*_, b_*i*_, c_*i*_, and d_*i*_ in the same stratum.

Lastly, the magnitude of confounding (CM) was calculated using the following formula,


(5) $$M\,a\,g\,n\,i\,t\,u\,d\,e\,o\,f\,c\,o\,n\,f\,o\,u\,n\,d\,i\,n\,g\,\left(C\,M\right)=\frac{O\,R_{c\,r\,u\,d\,e}-O\,R_{M\,H}}{O\,R_{M\,H}}$$

where OR_MH_ represents the adjusted odds ratio obtained using the Mantel-Haenszel formula (Kuritz et al., 1988) and OR_*crude*_ represents the crude (unadjusted) odds ratio.

## Results

From the TBIMS national database, we first grouped the subjects into mild, moderate, and severe TBI groups based on the GCS scores collected at the time of admission to the hospital (see section “Materials and methods”). Out of the 17,932 subjects in the TBIMS national database, 4,915 subjects belong to the mild category, 2,642 subjects belong to the moderate group and 3,007 subjects are severe TBI subjects. The remaining subjects were either chemically sedated or paralyzed or intubated or their GCS score was missing or unknown. Out of the 10,564 subjects from whom the GCS score was measured, 46.5 % belong to the mild category. We then computed the mean values of age at onset, GCS, and post-traumatic amnesia (PTA) for mild TBI subjects. The average age at injury onset for subjects with mild TBI is 55.47 ± 0.28 (mean ± sem) years. The mean values of PTA and GCS for the same group are 10.38 ± 0.21 days, 14.6 ± 0.007 respectively.

### Prevalence of comorbidities at 5 years following mild TBI

We next sought to understand the prevalence of various comorbidities at 5 years following the index TBI. The presence or absence of various medical and behavioural comorbidities was collected through a patient survey modelled based on NHANES (Center for Disease Control, National Center for Health Statistics, 1999). The 26 comorbidities that were covered in the NHANES interview were grouped under the following 8 categories: cardiovascular, neurological, musculoskeletal and rheumatologic, ophthalmological, endocrine, gastrointestinal, respiratory and psychiatric/mental health (Table 1). For each condition, we estimated the prevalence by counting the total number of subjects with the condition at the same time or after acquiring the index TBI and dividing this number by the total number of subjects who responded to the question (those with the presence and absence of the condition). We did the same to obtain the numbers for the prevalence of each condition with onset before acquiring TBI.

**TABLE 1:** Prevalence of psychiatric and medical comorbidities for mild TBI patients at 5-year follow-up time point.

Comorbidity	Before TBI		Same time or after TBI	
	***X*/*N***	**%**	***X*/*N***	**%**
**Cardiovascular**				
Hypertension	77/228	33.77	32/228	14.04
Congestive heart failure	11/226	4.87	5/226	2.21
Myocardial infarction	17/227	7.49	4/227	1.76
Other heart conditions	20/225	8.89	13/225	5.78
Stroke	9/226	3.98	13/226	5.75
High blood cholesterol	50/224	22.32	23/224	10.27
**Endocrine**				
Diabetes	33/226	14.60	13/226	5.75
**Gastrointestinal**				
Liver disease	10/228	4.39	7/228	3.07
**Musculoskeletal and Rheumatologic**				
Rheumatoid arthritis	8/224	3.57	6/224	2.68
Osteoarthritis	26/225	11.56	14/225	6.22
Chronic pain	26/223	11.66	35/223	15.70
**Neurologic**				
Sleep disorder	20/223	8.97	19/223	8.52
Movement disorder	1/224	0.45	2/224	0.89
Dementia	6/223	2.69	11/223	4.9
**Ophthalmologic**				
Cataracts	26/224	11.61	21/224	9.38
**Psychiatric/Mental health**				
Alcoholism	15/224	6.70	2/224	0.89
Drug addiction	10/223	4.48	3/223	1.35
Anxiety	29/223	13.00	36/223	16.14
Obsessive-compulsive disorder	2/223	0.089	3/223	1.35
Panic attacks	12/224	5.36	19/224	8.48
Post-traumatic stress disorder	8/224	3.57	25/224	11.16
Depression	35/223	15.70	35/223	15.70
Bipolar disorder	7/224	3.13	6/224	2.68
Attention deficit disorder/Attention deficit hyperactivity disorder	13/224	5.80	7/224	3.13
**Respiratory**				
Emphysema	15/228	6.58	9/228	3.95
Pneumonia	17/224	7.60	13/224	5.80

‘X’ represents the number of subjects with a specific comorbidity (before, same time or after TBI) and ‘N’ represents the total number of subjects with the presence and absence of the comorbidity.

The final number of mild TBI subjects that took part in the NHANES survey at 5-years post injury is 223-228. Out of this, 149-153 belong to the older cohort of patients and 69-71 belong to the younger cohort. 163-166 are males and 59-62 are female subjects. The prevalence of all 26 comorbidities for individuals with mild TBI at 5-year follow-up time point is given in Table 1. Using this data, we compiled a list of the top 10 comorbidities ranked according to their prevalence (at the same time or after TBI) at 5-year follow-up time point for subjects with mild TBI (Table 2). All further analyses in the study (old vs young and female vs male subjects) were performed for this list of highly prevalent comorbidities on subjects with mild TBI. A close inspection of Table 2 reveals that individuals with mild TBI are confronted with psychiatric conditions which include anxiety, depression, post-traumatic stress disorder (PTSD), and panic attacks (PA) at 5 years post injury. In addition to psychiatric comorbidities, cardiovascular comorbidities (hypertension (HT) and high blood cholesterol (HBC)) were also prevalent among subjects with mild TBI in the TBIMS national database. Lastly, the list includes other comorbidities like chronic pain (CP), osteoarthritis (OA), cataracts, and sleep disorder (SD). From these results, it becomes clear that subjects with mild TBI are confronted with a myriad of health-related issues at 5 years following the injury.

**TABLE 2:** Summary of top 10 most prevalent comorbidities for mild TBI subjects at 5-year follow-up.

Comorbidity	Prevalence (%)
Anxiety	16.14
Depression	15.7
Chronic pain	15.7
Hypertension	14.04
Post-traumatic stress disorder	11.16
High blood cholesterol	10.27
Cataracts	9.38
Sleep disorder	8.52
Panic attacks	8.48
Osteoarthritis	6.22

### Prevalence of comorbidities in young and old subjects

We next sought to determine if the prevalence of the top 10 medical and mental health comorbidities at the same time or after TBI differ between young and old subjects with mild TBI. Subjects were classified as young or old based on an age threshold of 50 years (see section “Materials and methods”). We asked the question if older subjects in the cohort exhibited increased odds of developing a particular set of comorbidities compared to younger subjects. To do this, we computed the OR for the top 10 prevalent comorbidities (compiled in the previous step) and established its significance using the Fisher’s-Exact test (see section “Materials and methods”). Our results indicate that psychiatric comorbidities (depression, anxiety, PTSD, and PA) exhibit an increased prevalence in the younger compared to an older cohort of subjects at the same time or after TBI. However, statistical significance was obtained only for the odds ratio of depression (OR = 0.37 and *p* = 0.026, Table 3). The prevalence of all other comorbidities (rheumatologic, ophthalmological, and cardiovascular) at the same time or after TBI was higher in the older cohort compared to the younger cohort. For example, subjects with age at follow-up > 50 years were 9.79 times more likely to experience OA compared to subjects with age at follow-up <= 50 years. Altogether, our results reveal a unique pattern of comorbidity dynamics in younger vs older individuals with mild TBI.

**TABLE 3:** Comparison of the prevalence of comorbidities according to the age of the subjects at 5-year follow-up.

Comorbidity	>50 years at 5-year follow-up		<= 50 years at 5-year follow-up		OR [CI]	*p*-value
	***X*/*N***	**%**	***X*/*N***	**%**		
Anxiety	22/149	14.76	14/70	20	0.71 [0.34–1.47]	0.46
Depression	17/150	11.33	17/69	24.63	0.37 [0.17–0.8]	0.016
Chronic pain	21/150	14	13/69	18.8	0.69 [0.33–1.47]	0.45
Hypertension	27/153	17.6	5/71	7.04	2.91 [1.16–7.33]	0.03
Post-traumatic stress disorder	14/150	9.3	11/70	15.71	0.52 [0.22–1.24]	0.2
High blood cholesterol	21/150	14	2/70	2.8	5.26 [1.46–18.9]	0.009
Cataracts	20/150	13.3	0/70	0	Inf	0.0001
Sleep disorder	14/150	9.3	5/69	7.2	1.31 [0.47–3.7]	0.79
Panic attacks	11/150	7.3	8/70	11.4	0.61 [0.23–1.64]	0.46
Osteoarthritis	13/151	8.6	1/70	1.4	9.79 [1.2–78.8]	0.018

‘*X*’ represents the number of subjects with a specific comorbidity (same time or after TBI) and ‘*N*’ represents the total number of subjects with the presence and absence of the comorbidity. OR, odds ratio; CI, confidence interval. The *p*-value was obtained using the Fisher’s - Exact test.

### Prevalence of comorbidities according to the biological sex of the subjects

We next sought to understand if the prevalence of comorbidities at the same time or after TBI was dependent on the biological sex of the subjects in the cohort. For this, we first computed the prevalence for the set of top 10 medical and psychiatric comorbidities at the same time or after TBI for male and female subjects in the cohort (Table 4). Since female subjects have a longer lifespan and tend to outlive men (Ginter and Simko, 2013), we reasoned that age could be a confounding factor in the association between sex and the prevalence of comorbidities. In order to control for confounding, we resorted to stratified analysis (see section “Materials and methods”) (Jager et al., 2008; Jose et al., 2008). Briefly, we created two strata of the confounder (age at follow-up): subjects with age at follow-up <= 50 years and subjects with age at follow-up <= 50 years. Stratum-specific odds ratio was computed for each stratum of the confounder. Based on the results from BDTT (Breslow and Day, 1980; Breslow, 1996), either effect modification or possible confounding was established. If effect modification was observed, stratum-specific odds ratios were reported while if confounding was observed unconfounded (adjusted) odds ratio (OR_MH_) (Kuritz et al., 1988; Jose et al., 2008; Tripepi et al., 2010) and magnitude of confounding (CM) was reported. Odds ratios reflect the odds of observing the comorbidity in female subjects compared to the odds in male subjects.

**TABLE 4:** Comparison of the prevalence of comorbidities according to the biological sex of the cohort at 5-year follow-up.

Comorbidity	Female		Male	
	***X*/*N***	**%**	***X*/*N***	**%**
Anxiety	11/59	18.6	25/164	15.2
Depression	7/60	11.67	28/163	17.2
Chronic pain	13/59	22.03	11/164	13.4
Hypertension	8/62	12.9	24/166	14.45
Post-traumatic stress disorder	10/60	16.67	15/164	9.1
High blood cholesterol	5/60	8.33	18/164	10.97
Cataracts	7/60	11.67	14/164	8.5
Sleep disorder	4/60	6.67	15/163	9.2
Panic attacks	6/60	10	13/164	7.9
Osteoarthritis	7/61	11.47	7/164	4.3

‘*X*’ represents the number of subjects with a specific comorbidity (same time or after TBI) and ‘*N*’ represents the total number of subjects with the presence and absence of the comorbidity.

Strong effect modification was observed for comorbidities such as depression, CP, SD and OA (*p* < 0.005 in the BDTT). Female subjects with age at follow-up <= 50 years exhibited decreased odds for depression compared to male subjects at 5-year follow-up (OR_2_ = 0.18, Table 5). Additionally, female subjects with age at follow-up > 50 years exhibited increased odds of developing CP compared to male subjects at 5-year follow-up (OR_1_ = 2.84, Table 5).

**TABLE 5:** Comorbidities for which effect modification was observed in female vs male subjects stratified by age at follow-up of 5-years.

Comorbidity	Prevalence in S1 (%)		Prevalence in S2 (%)		OR_1_ [CI]	OR_2_ [CI]	*p*_BDTT_	*p*_1_	*p*_2_
	**F**	**M**	**F**	**M**					
Depression	13	10	7	29	1.34 [0.56–3.23]	0.18 [0.08–0.44]	0.001	0.66	7.4e-5
Chronic pain	24	10	15	20	2.84 [1.28–6.31]	0.71 [0.34–1.47]	0.01	0.01	0.46
Sleep disorder	9	9	0	9	1 [0.38–2.6]	0	8e-3	1	0.003
Osteoarthritis	15	6	0	2	2.76 [1.03–7.45]	0	0.036	0.06	0.5

S1 represents stratum 1 (age at follow-up of 5 years > 50 years), S2 represents stratum 2 (age at follow-up of 5 years <= 50 years), OR_1_ and OR_2_ represent the odds ratio of female vs male subjects in S1 and S2 respectively, p_BDTT_ represents the *p*-value obtained using the Breslow-Day-Tarone test (BDTT), *p*_1_ and *p*_2_ represents the *p*-value of odds ratio in S1 and S2 respectively obtained using Fishers-Exact test. F, female; M, male; CI, confidence interval.

Possible confounding was observed for anxiety, HT, PTSD, HBC, and PA (Table 6). However, the magnitude of confounding was less than 10% for the above-mentioned comorbidities except for HBC. Female subjects demonstrated increased odds of developing PTSD compared to male subjects at 5-year follow-up (OR_MH_ = 1.88, Table 6). Our results, therefore, revealed interesting patterns in the association between biological sex and the prevalence of comorbidities post mild brain trauma.

**TABLE 6:** Comorbidities effect modification was not observed in female vs male subjects stratified by age at follow-up of 5-years.

Comorbidity	Prevalence in S1 (%)		Prevalence in S2 (%)		*p*_BDTT_	OR_crude_	OR_MH_	*p*_MHT_	CM
	**F**	**M**	**F**	**M**					
Anxiety	18	13	21	20	0.54	1.33	1.23	0.43	8.35
Hypertension	15	18	7	7	0.75	0.92	0.86	0.64	6.5
PTSD	15	7	21	14	0.55	2.07	1.88	0.03	10
High blood cholesterol	11	15	0	4	0.11	0.7	0.54	0.12	30.5
Panic attacks	9	7	14	11	0.99	1.28	1.31	0.41	-2.9

S1 represents stratum 1 (age at follow-up of 5 years > 50 years), S2 represents stratum 2 (age at follow-up of 5 years <= 50 years), OR_1_ and OR_2_ represent the odds ratio in S1 and S2 respectively, *p*_BDTT_ represents the *p*-value obtained using the Breslow-Day-Tarone test (BDTT), OR_crude_ represents the crude (unadjusted) odds ratio, OR_MH_ represents the adjusted (unconfounded) odds ratio computed using the Mantel-Haenszel formula, *p*_MHT_ represents the *p*-value obtained using Mantel-Haenszel test (MHT) and CM represents the magnitude of confounding. F, female; M, male.

## Discussion

We studied the longstanding comorbidities of acquiring mild TBI using patient data from the TBIMS national database. We did so by computing the prevalence of medical and mental health comorbidities at 5 years post index TBI. Our secondary aim is to understand how the prevalence of various comorbidities post mild TBI is influenced by a subset of patient demographics (sex, and age). Our analysis indicates the presence of interesting dynamics in the development of medical and mental-health comorbidities post TBI which could be useful to devise foresighted programs for the patient community. Our work hopes to contribute to the sparse body of literature concerning the prevalence of various comorbidities post mild TBI.

The findings in this study are similar to those that have been previously reported in the literature. A number of studies have reported that individuals with TBI exhibit an increased risk of developing psychiatric problems (Schwarzbold et al., 2008; Bryant et al., 2010; Bryant, 2011; Gould et al., 2011; Mallya et al., 2015; Stein et al., 2019) and demonstrate an impaired cognitive and oculomotor function (Dean and Sterr, 2013; Danna-Dos-Santos et al., 2018) compared to the general population. In our study, the most commonly manifested psychiatric disorders for individuals with mild TBI were anxiety and depression. Other frequently reported psychiatric conditions were PTSD, and PA. The prevalence of obsessive-compulsive disorder (OCD), bipolar disorder (BD), and attention deficit disorder/attention deficit hyperactivity disorder (ADD/ADHD) following TBI were far less common compared to other psychiatric conditions in our analysis. Confirming our results, depression, and generalized anxiety disorder were the two most common psychiatric conditions reported in a cohort of subjects with mild TBI from trauma centres in Australia (Bryant et al., 2010). Similarly, an increased risk prevalence of depression (and PTSD) was reported in a previous prospective study (Stein et al., 2019) of more than 1,000 individuals with mild TBI. Increased prevalence of anxiety and comorbid anxiety with depression have also been reported following TBI in the same study (Stein et al., 2019). These results have enormous clinical implications for the mild TBI community in post-traumatic disease management. For example, foresighted clinical programs could be implemented to screen the presence of depression, anxiety, and PTSD at multiple time points post acquiring mild TBI. Further, relatives of mild TBI survivors could be involved to watch out for symptoms of the above-mentioned psychiatric conditions in their family members.

We found that PTSD is highly prevalent in individuals with mild TBI (Table 2) and more so in female subjects (Tables 4, 6). Interestingly, in a previous TBIMS study (Hammond et al., 2019) that quantified the prevalence of comorbidities in individuals with moderate-severe TBIs, PTSD did not find itself in the list of top 10 comorbidities that were highly prevalent. Although the existence of PTSD after both mild (Middelboe et al., 1992; Bryant and Harvey, 1998; Bryant, 2011) and severe (Mcmillan, 1991, 1996; Bryant, 1996, 2011; Bryant et al., 2000) forms of TBI has been established in the literature, it’s possible that stronger PTA and loss of consciousness could retard the encoding of trauma-related experiences and prevent them from being re-experienced later (Bryant, 2011; Mallya et al., 2015). As a consequence, the risk of developing PTSD could be higher in mild cases of TBI compared to severe ones (Bryant, 2011; Mallya et al., 2015). Confirming this observation, a few studies have reported that the risk of developing PTSD is elevated in mild TBI compared to severe cases (Bombardier et al., 2006; Zatzick et al., 2010; Mallya et al., 2015).

With respect to cardiovascular comorbidities, we find that HT and HBC are quite common after mild TBI (Tables 1, 2). In line with our results, there are a few studies in the literature that associate TBI with an increased risk of cardiovascular comorbidities (Ahmadi et al., 2015; Wang et al., 2018; Nyam et al., 2019; Turner et al., 2021; Izzy et al., 2022). In a longitudinal study of individuals with TBI up to 10 years post injury, the authors found that cardiovascular and neurological comorbidities (Izzy et al., 2022) are highly prevalent in mild TBI as well as moderate-severely injured subjects. According to another study (Nyam et al., 2019) involving 16211 subjects who were followed up to 5 years post injury, TBIs are associated with an increased risk of developing cardiovascular comorbidities. In addition to that, TBIs are associated with an elevated risk of developing cardiovascular conditions such as arrhythmias and cardiomyopathies, and atherosclerosis (Ahmadi et al., 2015; Wang et al., 2018).

We also determined if the prevalence of comorbidities at the same time or after TBI exhibits differential patterns as a function of sex and the age of the participants at follow-up. Younger subjects (<= 50 years at follow-up) in our study were prone to developing depression compared to older participants who exhibited increased odds of developing cardiovascular (HT and HBC) comorbidities, cataracts, and OA (Table 3). Similar results were also seen in a previous study (Chan et al., 2017) where the younger cohort (<65 years old) was characterized by a high prevalence of psychiatric comorbidities whereas the older cohort (>65 years old) was characterized by the presence of cardiovascular, metabolic, and endocrine-related comorbidities. Such differential presence of comorbidities post TBI was also confirmed in a previous TBIMS study (Hammond et al., 2019).

With respect to the association between sex and prevalence of comorbidities, our results revealed that for a certain set of comorbidities, the association depends on the age at follow-up of the participants (effect modification) (Tables 5, 6). While depression was highly prevalent in young males compared to young females, the latter group exhibited increased odds of developing post-traumatic chronic pain (Table 5). Also, female subjects with mild TBI were more prone to developing PTSD compared to their male counterparts (Table 6). Women in general are more susceptible to developing PTSD (Olff, 2017), musculoskeletal conditions like OA (Zhang and Jordan, 2010), and anxiety disorders (McLean et al., 2011). In the TBI population, prior results have established that musculoskeletal (OA) and cardiovascular comorbidities are common in females compared to male subjects irrespective of age (Chan et al., 2017). Similarly, women TBI survivors are at an increased risk of developing PTSD, and depression (Kim et al., 2018) and were shown to exhibit an elevated severity in anxiety and depression (Mikolić et al., 2021).

### Limitations of the study and future work

The subjects in this study were those who received in-patient rehabilitation for mild TBI in one of the TBIMS centres (Traumatic Brain Injury Model Systems Program, 2021). These subjects were typically characterized by the presence of intracranial pathology and/or PTA (Traumatic Brain Injury Model Systems Program, 2021). Sufficient caution should be exercised while generalizing the results of the study to the mild TBI population as a whole since this population might also comprise subjects who were treated in less sophisticated hospitals/outpatient clinics or may even include subjects who may not seek medical attention. Further, our aim in this study is not to establish any sort of causal relationship but to elucidate the most frequently manifested psychiatric and medical problems in individuals with mild TBI. Future studies could incorporate a control group of non-TBI subjects for comparison and establishing causal inference. Lastly, the study could potentially suffer from ascertainment bias as it’s possible that some comorbidities that were diagnosed post TBI could have been persistent and undiagnosed prior to acquiring the injury due to lack of medical attention or some other reasons.

Another potential limitation of our study could be related to smaller sample sizes at each follow-up time point. For example, even though the TBIMS database consists of information pertaining to 4,915 mild TBI subjects, only 223-228 subjects enrolled in the NHANES study at 5-year follow-up time period. This could become problematic especially when comparing the prevalence between different patient subgroups where sample size could further diminish. As the TBIMS national database expands in the future with an increasing number of subjects, the current analysis could be repeated to see if there is any effect of sample size on the prevalence and statistical significance. Lastly, future administration of patient surveys/questionnaires (NHANES) could be expanded to cover other medical conditions.

This study is a preliminary excursion into the various factors that influence the development of comorbidities post mild TBI. In the future, we intend to build upon this work to study pairwise disease co-occurrences at scale using graph networks and network analysis (Fotouhi et al., 2018; Ljubic et al., 2020; Lee and Park, 2021). In doing so, a larger avenue of analysis is opened to visualize and understand disease comorbidities as differences in network structures across scales (Fotouhi et al., 2018; Ljubic et al., 2020; Lee and Park, 2021). Comorbidities are understood as unique spatial organisations of statistically significant interconnected disease nodes with varying depths of connectedness (Fotouhi et al., 2018; Kim et al., 2018; Lee and Park, 2021). Network structural properties will be compared across time, TBI severity, and demographic conditions. The significance of this approach is an intuitive visualisation of diseases in terms of their co-occurrences, and clusters of diseases can be identified for unique TBI severity and demographic conditions that could inform proactive post-TBI healthcare. The current literature on using disease networks to study comorbidity relationships in subjects with TBI is sparse and we seek to contribute to that body of knowledge. Finally, as the incidence of mild TBI is frequent in the general population, future excursions of the work could include understanding the comorbidity dynamics as a function of the number of impacts experienced by the subjects.

## Conclusion

Our study indicates that individuals who have sustained mild TBI could develop serious long-term medical and psychiatric comorbidities which could affect their pace of recovery. The results of this study could motivate further investigation regarding the prevalence of health-related problems in the mild TBI population and encourage the development of foresighted programs and patient care for post-traumatic management of comorbidities. The findings reported in the paper have wider implications for prognosis, patient management, and treatment of various comorbidities after TBI.

## Data availability statement

Publicly available datasets were analyzed in this study. This data can be found here: https://www.tbindsc.org.

## Ethics statement

This study is secondary data analysis of a publicly available dataset. The original studies involving human participants were reviewed and approved by Krea Institutional Review Board. Written informed consent to participate in the original study was provided by the participants’ legal guardian/next of kin.

## Author contributions

SS: conceptualization, methodology, investigation, writing—review and editing. SC: Conceptualization, investigation, writing—review and editing. KP: investigation, writing—review and editing. KM: methodology, writing—review and editing. SKS: conceptualization, methodology, software, validation, formal analysis, investigation, resources, data curation, writing—original draft, writing—review and editing, visualization, supervision, project management, and funding acquisition. All authors contributed to the article and approved the submitted version.
